# Crocin, a plant-derived carotenoid, modulates microglial reactivity

**DOI:** 10.1016/j.bbrep.2017.09.007

**Published:** 2017-10-02

**Authors:** Mücella Arikan Yorgun, Khalid Rashid, Alexander Aslanidis, Charlotte Bresgen, Katharina Dannhausen, Thomas Langmann

**Affiliations:** Laboratory for Experimental Immunology of the Eye, Department of Ophthalmology, University of Cologne, D-50931 Cologne, Germany

**Keywords:** Crocin, Microglia, Retina

## Abstract

Microglia activation plays an important role in immune responses in the CNS including the retina. Crocin, a plant-derived carotenoid, has been reported to possess anti-inflammatory, anti-apoptotic and anti-oxidative capacity in models of retinal damage and degeneration. If these neuroprotective effects could be mediated by direct modulation of microglial cells is unclear. Here, we examined the direct effects of crocin on key functions and pro-inflammatory gene expression in lipopolysaccharide (LPS)-activated BV-2 microglia. We found that crocin stimulation strongly promoted filopodia formation and markedly increased microglial phagocytosis, two important parameters relevant for physiological microglia functions. Moreover, crocin significantly reduced gene expression of the pro-inflammatory markers IL6, CCL2, and iNOS in LPS-challenged BV-2 cells and potently blocked NO production in these microglia. The observed immunomodulatory effects of crocin were not mediated by general inhibition of NFkB nuclear translocation. Our findings indicate that many of the anti-inflammatory effects of crocin demonstrated in animal models of neuronal degeneration could be mediated by its direct effects on microglia homeostasis.

## Highlights

• Crocin promotes microglial filopodia formation.
• Crocin induces the phagocytic capacity of microglia.
• Crocin dampens LPS-induced pro-inflammatory gene expression and lowers nitric oxide production in microglia.

## 1 Introduction

Microglial cells are resident macrophages of the central nervous system including the retina and have important roles in retinal and neuronal homeostasis [1], [2]. In the “surveillance’’ state, they have a small soma with fine cellular processes and ramified morphology, which actively scan their environment [3]. Under pathological conditions microglia transform to “reactive” states, which involves changes in morphology into an amoeboid shape, their migration to injury site and the release of pro-inflammatory and cytotoxic factors [4]. While microglial activation may have a protective role in the maintenance of retinal integrity [5], microglial may even actively contribute to retinal degenerative diseases [6]. Consequently, chronic activation of these cells has been documented in various neurodegenerative diseases of the retina, including age-related macular degeneration [7], inherited photoreceptor dystrophies [8], and glaucoma [9]. Therefore, modulation of microglial reactivity emerged as therapeutic strategy to treat retinal degenerative diseases [10].

Recent years witnessed a growing interest in the discovery of natural compounds that have an impact on neuroinflammatory processes. Among these natural immunomodulators, saffron has been used worldwide in traditional medicine [11]. Saffron and its active ingredients mainly crocin, crocetin and safranal exert anti-proliferative, anti-tumor, anti-inflammatory, anti-oxidant, anti-apoptotic, and hepatoprotective effects [12]. Administration of saffron components in different models of retinal damage and degeneration showed neuroprotective effects [13], [14]. Likewise, safranal could slow down photoreceptor degeneration in the P23H rat model of retinitis pigmentosa, reflected by improved a- and b-wave amplitudes in electroretinographic recordings and a preserved vascular network [14]. Similarly, crocin and crocetin had protective effects against light induced photoreceptor degeneration in vitro and in vivo [15], [16], [17]. Moreover, crocin significantly prevented retinal ganglion cell apoptosis after retinal ischaemia/reperfusion injury [18]. There are also indications, that saffranal supplementation in patients with age-related macular degeneration (AMD) improved their retinal function as detected by increased focal electroretinograms (fERGs) amplitudes [19]. Despite these widely reported neuroprotective effects of crocin, it is unclear whether these beneficial effects may be mediated, at least partially, by modulation of microglial reactivity. In this study, we aimed to investigate the direct immune-modulatory effect of crocin in BV-2 microglial cells.

## 2 Materials and methods

### 2.1 Cell culture

BV-2 microglia were cultured in RPMI 1640 with 5% fetal calf serum (FCS) supplemented with 2 mM L-glutamine, 1% penicillin/streptomycin and 195 nM β-mercaptoethanol at 37 °C in a humidified atmosphere of 5% CO2 as previously described [20]. BV-2 cells were pre-incubated with 200 μM crocin or PBS as vehicle control in fresh medium, without FCS, for 30 min. Afterwards, the cells were stimulated with 50 ng/ml LPS, 200 μM crocin, or 50 ng/ml LPS + 200 μM crocin for 24 h. These stimulation conditions were adopted from preliminary experiments which revealed that 200 μM crocin was the most effective dose and had no cytotoxic effects (data not shown). 661 W photoreceptor-like cells were a gift from Prof. Muayyad Al-Ubaidi (Department of Cell Biology, University of Oklahoma Health Sciences Center, Oklahoma City, OK, USA), and the culture conditions have been described elsewhere [21].

### 2.2 Phalloidin-TRITC staining

BV-2 microglial cells were seeded on cover slips in six-well plates and cultivated and stimulated as described before. Afterwards cells were fixed with 4% formaldehyde, permeabilized with 0.1% Triton X-100, and F-actin was fluorescently labeled using 0.1 μg/ml Phalloidin-TRITC (Sigma-Aldrich). Nuclei were stained using DAPI, and the cover slips were mounted with fluorescent mounting medium (Dako Cytomation). Photomicrographs were taken with an AxioImager. M2 plus ApoTome2 microscope (Carl Zeiss). Quantitative scoring of microglial ramification was performed as described previously using a grid-cross analysis [6].

### 2.3 Phagocytosis assay

Phagocytosis assay 661 W photoreceptor cells were starved for two weeks with serum deprivation, harvested and fluorescently labeled using CellTracker CM-DiI (Invitrogen, Carlsbad, CA, USA). BV-2 cells were left to adhere on coverslips overnight and pre-treated with PBS or crocin for 30 min. Afterwards microglial cells were cultivated with or without LPS for further 24 h. In the following 400 μl labeled apoptotic photoreceptor material was added. After a further cultivation period of 6 h, microglial cells were washed and nuclei were stained with DAPI. Fluorescence micrographs were then taken and ImageJ software (National Institutes of Health, Bethesda, MD, USA) was used to determine the fluorescent intensities. After subtracting background intensities from both DsRed (phagocytosed apoptotic photoreceptor) and DAPI (total microglia cell number) signals, the Phagocytosis Index was determined by dividing the corrected DsRed signal by the corrected DAPI signal and giving these numbers in percent [20].

### 2.4 RNA isolation and real-time RT-PCR

Total RNA was extracted from microglia cells according to the manufacturer's instructions using the NucleoSpin® RNA Mini Kit (Macherey-Nagel, Dueren, Germany). RNA was quantified spectrophotometrically using a NanoDrop 2000 (Thermo Scientific) and then stored at −80 °C. First-strand cDNA synthesis was performed with the RevertAid™ H Minus First strand cDNA Synthesis Kit (Fermentas, Schwerte, Germany). Amplifications of 50 ng cDNA were performed with an ABI7900HT machine (Applied Biosystems, Carlsbad, CA, USA) in 10 μl reaction mixtures containing 1 × TaqMan Universal PCR Master Mix (Applied Biosystems), 200 nM of primers and 0.25 μl of dual-labeled probe (Roche ProbeLibrary, Roche Applied Science, Basel, Switzerland). The reaction parameters were as follows: 2 min 50 °C hold, 30 min 60 °C hold and 5 min 95 °C hold, followed by 45 cycles of 20 s 94 °C melt and 1 min 60 °C anneal/extension. Primer sequences and Roche Library Probe numbers were as follows: CCL2, forward primer 5′-catccacgtgttggctca-3′, reverse primer 5′-gatcatcttgctggtgaatgagt-3′, probe #62; IL6, forward primer 5′-gatggatgctaccaaactggat-3′, reverse primer 5′-ccaggtagctatggtactccaga-3′, probe #6; iNOS, forward primer 5′-ctttgccacggacgagac-3′, reverse primer 5′- tcattgtactctgagggctga-3′, probe #13; Measurements were performed in triplicates. ATPase, forward primer 5′-ggcacaatgcaggaaagg-3′, reverse primer 5′-tcagcaggcacatagatagcc-3′, probe #77. ATPase expression was used as reference gene and the results were analyzed with the ABI sequence detector software version 2.4 using the ΔΔCt method for relative quantification [6].

### 2.5 Nitrite assay

The nitrite concentration in culture supernatants was determined as an indicator of nitric oxide (NO) production using the Griess reagent system (Promega). 50 μl cell culture supernatants were incubated with an equal volume of Griess reagent in each well of a translucent 96-well plate. After incubation for 30 min at room temperature, the absorbance was read at 540 nm on an Infinite F200 pro plate reader (Tecan). Nitrite concentrations for each sample were calculated from a sodium nitrite standard curve.

### 2.6 Immunocytochemistry

BV-2 cells were seeded on sterile cover slips and were cultivated and stimulated as described before. Afterwards cells were fixed with 4% formaldehyde, rehydrated with PBS, and subsequently blocked with buffer, containing 10% goat serum and 0.3% Triton X-100 for 30 min. The cells were then incubated with primary antibody against p65 subunit of NF-κB diluted in PBS containing 2.5% goat serum and 0.1% Triton X-100 for 1 h at room temperature. Afterwards slides were washed with PBS and incubated with goat anti-rabbit Alexa-594 (A-11012, Life Technologies) for further 30 min. Nuclear DNA was stained with 4′, 6-diamidino-2-phenylindole (DAPI). Cover slips were mounted with fluorescent mounting medium (Dako Cytomation, Hamburg, Germany), and fluorescence photomicrographs were taken with an AxioImager. M2 plus ApoTome2 microscope (Carl Zeiss, Oberkochen, Germany). NF-κB translocation was determined measuring the mean fluorescence intensity of the nuclei and cytoplasmic areas using Image J. A nucleus/cytoplasma signal ratio above 1 indicates nuclear localization.

### 2.7 Statistical analysis

Real-time RT-PCR data, nitrite secretion and phagocytosis were analyzed with ANOVA and Tukey's multiple comparisons test. *P* < 0.05 was considered as statistically significant.

## 3 Results and discussion

### 3.1 Crocin promotes microglial filopodia formation

**Fig. 1:**
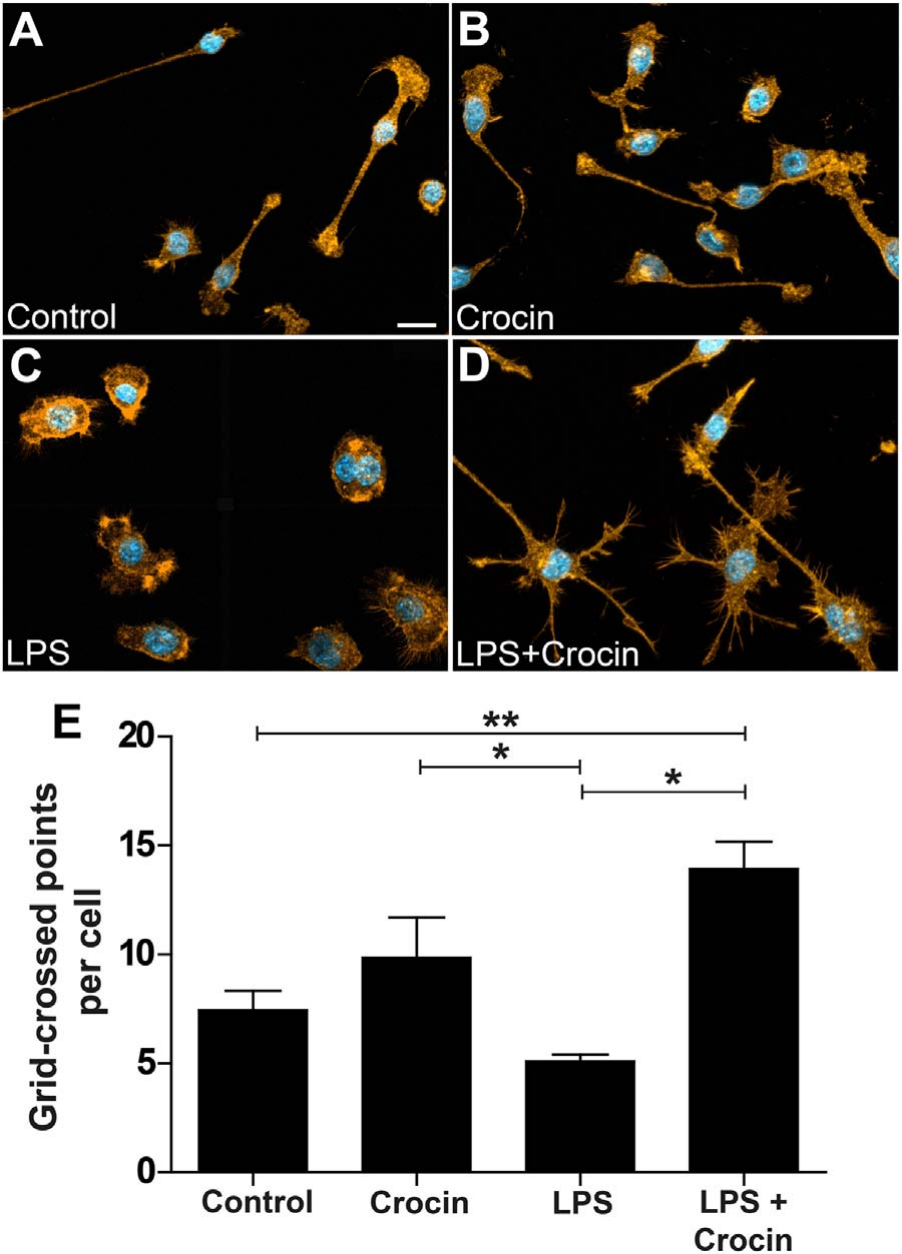
**Crocin promotes microglial filopodia formation**. Representative images of Phalloidin-TRITC/DAPI labeled BV-2 microglial cells showing morphological changes in response to stimulation with vehicle (A), 200 µM crocin (B), 50 ng/ml LPS (C), and 50 ng/ml LPS plus 200 µM crocin (D). Cells were pre-treated with 200 μM crocin for 30 min, followed by stimulation with 50 ng/ml LPS for further 24 h. Scale bar = 20 µm. (E) Quantification of microglial ramification was performed using a grid-cross counting method [6]. Data show mean ± SEM (n = 13–18 cells/group in three independent images), **P < 0.01 for LPS + crocin- versus control, *P < 0.05 for LPS + crocin- versus LPS alone, and *P < 0.05 for LPS versus crocin.

We first studied whether crocin can directly influence microglial morphology and filopodia formation. A high number of filopodia with many branching points is commonly regarded as a sign of surveillance function, whereas a round amoeboid shape is a sign of reactivity. BV-2 cells were left untreated or treated with 50 ng/ml LPS, 200 µm crocin or both together and the f-actin cytoskeleton was stained using Phalloidin-TRITC (Fig. 1). Immunofluorescence analysis revealed that microglia had some filopodia in control conditions (Fig. 1A) and despite some tendency for more filopodia with crocin treatment, the change was not statistically different (Fig. 1B, E). Incubation with LPS caused a complete rounding of cells (Fig. 1C), and the combined treatment with crocin reversed this amoeboid phenotype with a statistically increased level of ramification (Fig. 1D, E). These analyses clearly show that crocin directly regulates filopodia formation in LPS-treated microglia.

### 3.2 Crocin induces the phagocytic capacity of microglia

**Fig. 2:**
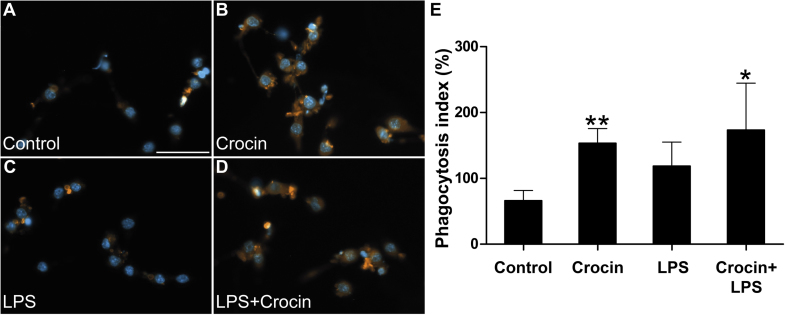
**Crocin enhances the phagocytic capacity of microglia.** Representative images showing phagocytic uptake of CM-DiI-stained apoptotic 661 W photoreceptor material into BV-2 cells treated with vehicle (A), 200 μM crocin (B), 50 ng/ml LPS (C), or 50 ng/ml LPS plus 200 μM crocin (D) for 6 h. (E) Bar graphs showing quantification of microglial phagocytosis as phagocytosis index in %. Data show mean ± SD (n = 9 cultures/group), **P < 0.01 for crocin versus vehicle-treated, *P < 0.05 for LPS + crocin- versus LPS alone. Scale bar = 50 µm.

We next tested the effect of crocin on the phagocytic capacity, an important physiological function of microglia [22]. Fluorescently labeled dying 661 W photoreceptor-like cells were used as cargo that mimics cell debris in the degenerating retina. BV-2 microglial cells stimulated with crocin displayed a significantly higher phagocytosis rate that control cells (Fig. 2A, B, F, *P* < 0.01). This effect of stimulated phagocytosis was also present in LPS-pretreated BV-2 cells (Fig. 2E, C, F, *P* < 0.044). These findings indicate that crocin promotes the ramified microglial phenotype with a high phagocytosis capacity. The exact mechanisms how crocin increases microglial phagocytosis are currently unknown. However, crocin-treated peritoneal mouse macrophages also showed increased yeast phagocytosis, corroborating our data [23].

### 3.3 Crocin dampens LPS-induced pro-inflammatory gene expression and lowers nitric oxide production in microglia

**Fig. 3:**
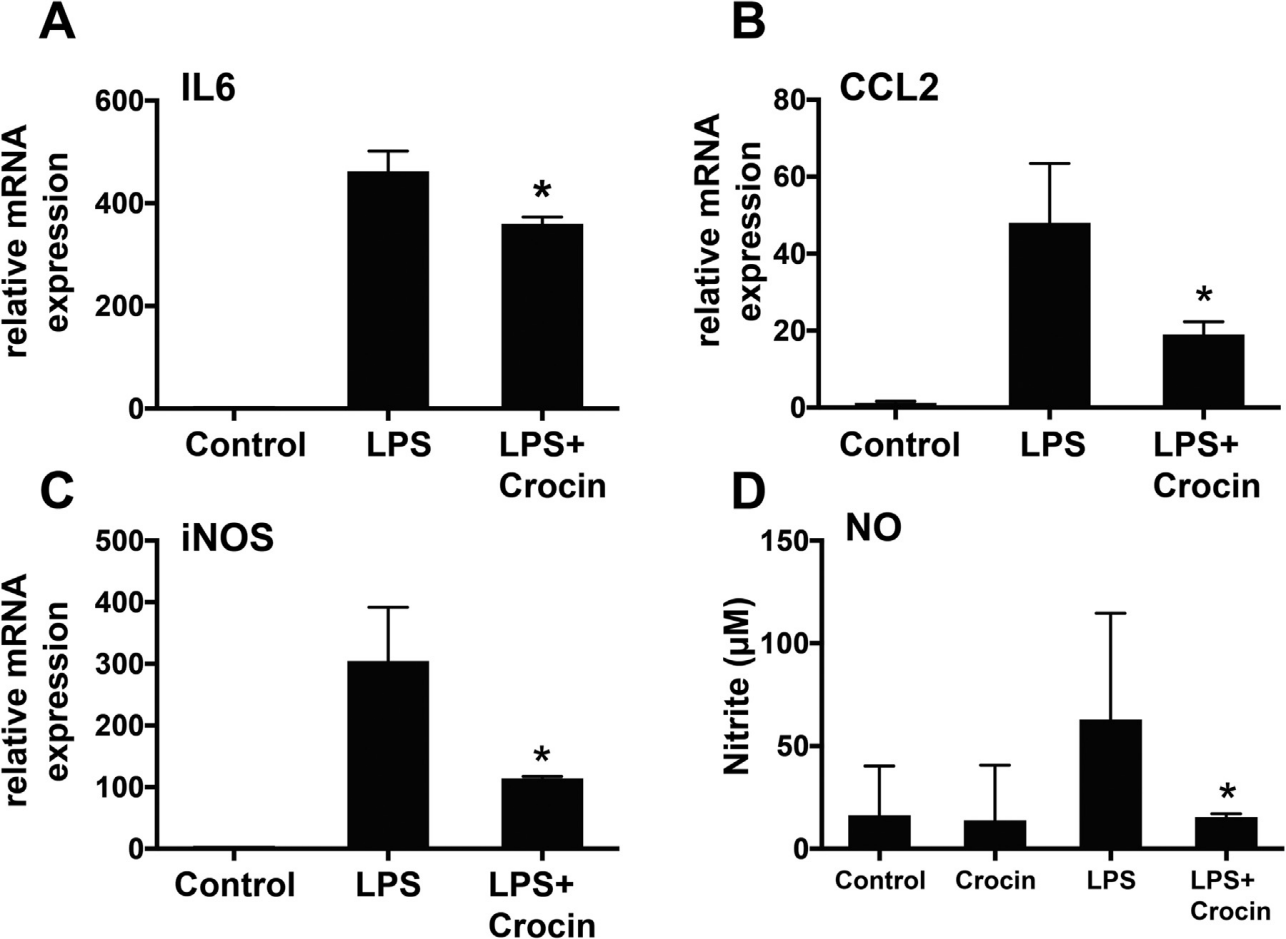
**Crocin dampens pro-inflammatory gene transcription and reduces microglial NO production.** BV-2 cells were either treated with PBS as a vehicle control or 200 μM crocin for 30 min before stimulation with 50 ng/ml LPS for further 24 h. IL6 (A), CCL2 (B), and iNOS (C) mRNA levels were determined by quantitative real-time PCR. Data show mean ± SD (n = 3 biological replicates, measured in triplicate). (D) The concentration of nitrite in the culture supernatants was measured by Griess reaction. Data show mean ± SD (n = 9 biological replicates). *P < 0.05 for LPS + crocin- vs. LPS-treated cells (A-D).

We then analyzed whether crocin can directly modulate pro-inflammatory gene expression in microglia. Interleukin 6 (IL6), CC-chemokine ligand 2 (CCL2) and inducible NO synthase (iNOS) were chosen as representative markers for molecular pathways involved in acute phase response, chemotaxis and oxidative burst, respectively. LPS strongly induced IL6 (Fig. 3A), CCL2 (Fig. 3B) and iNOS (Fig. 3C) in BV-2 microglia. Co-treatment with 200 µm crocin significantly reduced the LPS-induced gene transcription of IL6 (Fig. 3A, *P* < 0.021), CCL2 (Fig. 3B, *P* < 0.033), and iNOS (Fig. 3C, *P* < 0.025). To study whether this effect was also seen on a function level, the production and secretion of NO radicals was measured. Crocin treatment alone did not influence NO secretion from microglia (Fig. 3D). However, the LPS-induced production of NO was significantly diminished by the presence of crocin in the culture medium (Fig. 3D, *P* < 0.017). These observed effects are consistent with previous reports, showing that crocin can dampen LPS-induced production of pro-inflammatory IL1β and TNFα and NO in cultured rat brain microglial cells and RAW 264.7 macrophages [24], [25]. Taken together, these findings support the anti-inflammatory and immunomodulatory effects of crocin on microglial cells.

### 3.4 Crocin does not inhibit nuclear translocation of NFkB

**Fig. 4:**
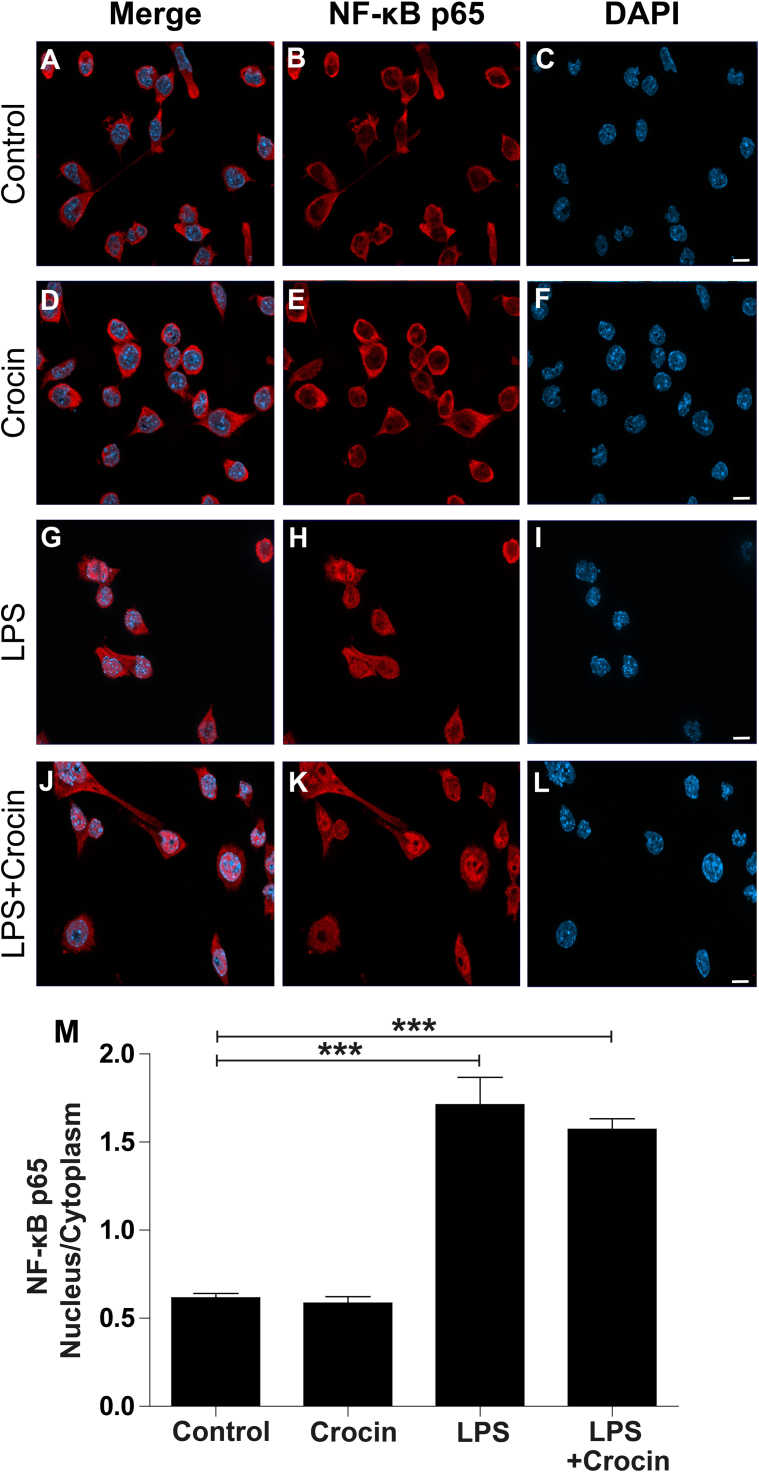
**Crocin does not inhibit nuclear translocation of NFkB.** Representative immunofluorescence stainings showing NFkB p65 subunit localization in BV-2 cells treated with vehicle (A-C), 200 μM crocin (D-F), 50 ng/ml LPS (G-I), and 200 μM crocin + 50 ng/ml LPS (J-L). The NFkB p65 subunit mainly resides in the cytoplasm of control and crocin treated BV-2 cells. Prominent nuclear NFkB p65 staining in both LPS (G-I) and LPS + crocin (J-L) stimulated cells, indicating that crocin does not directly interfere with NFkB p65 subunit translocation into the nucleus. (M) Quantification of nuclear NFkB p65 subunit translocation. Data show mean ± SEM (n = 42–85 cells). ***P < 0.001 for LPS versus control and LPS + crocin- vs. control.

Since NFkB is an important pro-inflammatory signaling pathway, we finally investigated the effect of crocin on the nuclear translocation of the NFkB p65 subunit. Immunostaining with a specific anti-NFkB p65 antibody showed that most protein resides in the cytosol of unstimulated microglia (Fig. 4A-C) and cells treated with crocin alone (Fig. 4D-F). The activation of microglia with LPS promoted the translocation of NFkB from the cytosol to the nucleus within minutes (Fig. 4G-I). Co-incubation with crocin did not significantly influence NFkB translocation (Fig. 4J-M), suggesting that crocin's immunomodulatory effects are not directly mediated by inhibition of the NFkB p65 pathway. These data are contrary to the study by Nam et al. demonstrating that both crocin and crocetin effectively reduced LPS-elicited NFkB activation in BV-2 microglia [24]. One explanation could be that we used direct immunostaining whereas Nam et al. indirectly measured NFkB-dependent luciferase activity [24]. Further analyses using Western Blotting of nuclear extracts are under way to clarify this discrepancy.
